# Concordance Between Clinical and Histopathological Diagnoses of Oral and Maxillofacial Lesions in Thailand: A 20-Year Retrospective Study

**DOI:** 10.4317/jced.62771

**Published:** 2025-07-01

**Authors:** Chanyanuch Teerawong, Tanyaaonann Chulaksanadecha, Thiyada Anansila, Punnapa Krasaetanont, Chaidan Intapa, Chalatip Chompunud Na Ayudhya

**Affiliations:** 1 Department of Oral Diagnosis, Faculty of Dentistry, Naresuan University, Phitsanulok, Thailand; 2 Faculty of Dentistry, Naresuan University, Phitsanulok, Thailand; 3 Faculty of Dentistry, Nation University, Chiang Mai, Thailand

## Abstract

**Background:**

Accurate diagnosis is crucial for effective treatment planning and management. This study aims to evaluate the concordance between clinical and histopathological diagnoses of oral biopsy specimens submitted to the Oral Pathology Unit, Faculty of Dentistry, Naresuan University, Thailand, over a 20-year period.

**Material and Methods:**

Histopathology reports from 2004 to 2023 were retrieved and reviewed. Data on age, gender, lesion location, clinical diagnosis, and histopathological diagnosis were collected. The concordance between clinical and histopathological diagnoses, as well as factors associated with the concordance rate, were analyzed.

**Results:**

Of 1,253 biopsied cases, a total of 1,148 were included in the study. These comprised 513 males (44.69%) and 635 females (55.31%), with an average age of 40.97 ± 20.70 years. The majority of lesions were reactive and inflammatory lesions (35.1%), followed by epithelial pathology (10.02%), and immune-mediated lesions (7.58%), respectively. Twenty-nine cases (2.53%) were malignant. Overall, clinico-pathological concordance was observed in 69.34% of cases. The highest and lowest concordance rates were noted in immune-mediated lesions and non-odontogenic cysts and tumors, respectively.

**Conclusions:**

There is considerable variability in concordance among different oral lesion types. The results of this study provide insights into the prevalence of oral lesions in a group of Thai population and offer a valuable database for clinicians in developing clinical diagnoses for oral lesions.

** Key words:**Concordance, Oral biopsy, Clinical diagnosis, Histopathological diagnosis.

## Introduction

Oral and maxillofacial lesions encompass a wide range of conditions, including ulcerations, cysts, benign tumors, and malignancies. These lesions can affect patients’ quality of life by impairing mastication, swallowing, and speech, often causing symptoms such as burning, irritation, and pain (1). While some lesions exhibit distinct characteristics and clinical features that facilitate diagnosis, many share similar clinical presentations, posing diagnostic challenges.

Although histopathological examination is considered the gold standard for the definitive diagnosis of oral and maxillofacial lesions (2), an initial clinical diagnosis plays a crucial role in provisional assessment and management of these conditions. An accurate clinical diagnosis guides appropriate investigations and treatment strategies tailored to the lesion’s severity and nature, thereby preventing unnecessary or delayed interventions. Conversely, an incorrect clinical impression may postpone treatment, potentially increasing morbidity and mortality rates while diminishing the patient’s quality of life, particularly for lesions with malignant potential (3). Furthermore, in such cases, pathologists rely on the clinical symptoms and detailed information provided by the dentists to establish a conclusive diagnosis (4). This highlights the importance of effective collaboration between clinicians and pathologists, as well as the clinician’s careful attention to critical diagnostic details.

Although several epidemiological studies have examined the concordance between clinical and histopathological diagnoses of oral and maxillofacial lesions (3,5-11), to the best of our knowledge, no study has investigated concordance rates among dental patients in Thailand. Therefore, this retrospective study aimed to evaluate the prevalence of oral lesions histopathologically diagnosed at the Faculty of Dentistry, Naresuan University, Thailand, over the past 20 years, as well as to assess the concordance rates between clinical and histopathological diagnoses. The findings of this study may help clinicians improve diagnostic accuracy, facilitate effective management decisions, and optimize patient care.

## Material and Methods

- Study Design and Sample

This retrospective study was approved by the Institutional Review Board of Naresuan University (NU-IRB-COE No. 007/2024). Biopsy records of specimens submitted to the Oral Pathology Unit, Faculty of Dentistry, Naresuan University, Thailand, from both institutional and external hospitals between 2004 and 2023 were retrieved and reviewed. The collected data included age, gender, lesion location (maxilla, mandible, buccal mucosa, gingiva, tongue, palate, upper and lower lips, and other sites such as the maxillary sinus and floor of the mouth), clinical diagnosis, and histopathological diagnosis.

Exclusion criteria included extraoral lesions, lack of a clinical diagnosis, and cases with inconclusive histopathological diagnoses. Multiple biopsies from the same patient and the same location were considered as a single lesion. The included lesions were then categorized into seven groups:

1. Epithelial pathology: Lesions that originate from or primarily involve the epithelial lining of the oral mucosa. These may be benign, premalignant, or malignant in nature.

2. Reactive and inflammatory lesions: Lesions that arise as a response to local irritation, trauma, or infection.

3. Immune-mediated lesions: Disoders caused by autoimmune or hypersensitivity reactions.

4. Bone pathology: Lesions that originate from or primarily affect the bony structures of the jaws. These may be developmental, inflammatory, or neoplastic.

5. Odontogenic cysts and tumors: Lesions derived from odontogenic epithelium or ectomesenchyme involved in tooth development.

6. Non-odontogenic cysts and tumors: Cysts and tumors that arise independently of odontogenic tissues but occur within the oral and maxillofacial region. These may originate from developmental, epithelial, mesenchymal, or salivary gland tissues.

7. Miscellaneous: Lesions or conditions that do not fit into the above categories.

- Determination of Diagnostic Concordance

The concordance between clinical and histopathological diagnoses was assessed. Histopathology reports were reassessed for specimen location, macroscopic and microscopic findings, definitive diagnosis, and any additional comments or suggestions from the pathologist.

Lesions were classified based on the relationship between the clinical diagnosis and the histopathological diagnosis as follows:

• Concordant:

The histopathological diagnosis confirmed the clinical diagnosis, either by:

o An exact match in terminology or diagnostic entity, or

o Sufficient alignment between the clinical impression and histopathological findings based on diagnostic standards.

(e.g., a clinical diagnosis of leukoplakia with a histopathological diagnosis of epithelial hyperplasia, with or without epithelial dysplasia).

• Discordant:

The histopathological diagnosis did not confirm the clinical diagnosis due to one of the following:

o A completely different diagnostic entity was identified histologically, or

o The clinical and histopathological diagnoses could not be reliably compared or correlated.

- Statistical Analyses

Data were analyzed using the Statistical Package for Social Sciences (SPSS), version 24.0 (SPSS Inc., Chicago, IL, USA). A descriptive statistical analysis of all variables was conducted. The Chi-square test was applied to examine the differences in categorical variables across the groups. A *p-value* of less than 0.05 was considered statistically significant.

## Results

Out of 1,253 biopsied cases, a total of 1,148 were included in the study. The overall concordance rate between clinical and pathological diagnoses was 69.34%.

- Age and Gender

This study comprised 513 males (44.69%) and 635 females (55.31%), with a mean age of 40.97 ± 20.7 years. The age distribution peaked in the 41–60-year age range. Among the lesion categories, epithelial pathology had the highest mean age of 56.02 years, whereas odontogenic cysts and tumors had the lowest mean age of 32.54 years. Age was significantly associated with the occurrence of bone pathology (*p* < 0.001), epithelial pathology (*p* = 0.004), odontogenic cysts and tumors (*p* < 0.001), reactive and inflammatory lesions (*p* < 0.001), and miscellaneous lesions (*p* = 0.002) ( Table 1). Additionally, gender showed a significant association with the occurrence of odontogenic cysts and tumors (*p* = 0.001) ( Table 1). However, no significant correlation was found between age group or gender and the concordance between clinical and histopathological diagnoses of the lesions (*p* = 0.841 and *p* = 0.321, respectively) ( Table 2, Table 3).

- Groups of Oral Lesions

Among the 7 lesion categories, the majority of cases were reactive and inflammatory lesions (35.10%), followed by odontogenic cysts and tumors (32.93%), and epithelial pathology (10.02%). The most frequently diagnosed lesions were radicular cysts (9.41%), dentigerous cysts (7.84%), and fibromas (6.71%), respectively. Detailed frequency and concordance rates for each lesions are presented in Table S1, (Supplement 1) (http://www.medicinaoral.com/medoralfree01/aop/jced_62771_s01.pdf).

The highest concordance between clinical and histopathological diagnoses was observed in immune-mediated lesions (93.1%), while the lowest was found in non-odontogenic cysts and tumors (50%). A significant correlation was identified between clinicopathological concordance and the following lesion groups: reactive and inflammatory lesions (*p* < 0.001), immune-mediated lesions (*p* = 0.004), odontogenic cysts and tumors (*p* < 0.001), non-odontogenic cysts and tumors (*p* = 0.013), and miscellaneous lesions (*p* = 0.018) ( Table 1).

- Location

Lesions were most commonly located in the mandible (27.87%), followed by the maxilla (25.26%), gingiva and alveolar gingiva (16.64%), and buccal mucosa (13.06%). Less frequently affected sites included the maxillary sinus and floor of mouth. A significant association was found between lesion location and the concordance between clinical and histopathological diagnoses (*p* = 0.001), suggesting that the accuracy of clinical diagnosis may vary depending on the site of the lesion ( Table 4).

- Benign versus Malignant Lesions

Out of the total cases, 1,119 lesions (97.47%) were benign, while 29 (2.53%) were malignant. Squamous cell carcinoma was the most prevalent malignancy, representing 75.9% of the malignant cases. The concordance between clinical and histopathological diagnoses for malignant lesions was 55.17% (Table 5). The frequency, concordance rates, and observations on misdiagnosis for each malignant lesion are provided in Table S2 (Supplement 2) (http://www.medicinaoral.com/medoralfree01/aop/jced_62771_s02.pdf).. Age was significantly associated with the occurrence of malignant lesions (*p* < 0.001), and females were more likely to be diagnosed with malignant neoplasms than males (*p* = 0.024) ( Table 5).

## Discussion

The concordance between clinical and histopathological diagnoses of oral lesions in Thailand has not been previously established. Understanding the correlation between clinical and histopathological diagnoses, as well as the prevalence of oral lesions, is essential for enhancing dentists’ awareness, improving diagnostic accuracy, and ensuring effective treatment approaches. To the best of our knowledge, the present study is the first to evaluate concordance rates and identify factors associated with diagnostic discrepancies between clinical and histopathological diagnoses in Thai patients. Herein, provisional clinical diagnoses and definitive histopathological diagnoses had an agreement rate of 69.34%, falling within the range reported in previous studies from other countries, which have documented concordance rates between 50.6% and 94.4% (3,5-10). The type and location of lesions were found to be significantly correlated with the accuracy of clinical and histopathological diagnoses.

The prevalence and epidemiological profiles of oral lesions vary across different regions of the world due to demographic and geographic differences in the studied populations (12,13). In this study, the majority of patients were women, with a male-to-female ratio of 0.8:1, and the most common age group was between 41 and 60 years. These findings align with previous epidemiological studies on oral lesions in Thailand by Lam-ubol *et al*. (14) and Rodphon *et al*. (12) which demonstrate a predilection toward middle-aged females. This trend may be attributed to gender differences in care-seeking behavior and awareness of oral health (15).

Some oral lesions, such as oral lichen planus (OLP) and oral squamous cell carcinoma (OSCC), are more commonly associated with specific age groups and genders (16,17). Consistent with this, we found that epithelial pathologies, including oral leukoplakia and OSCC, had the highest mean age, whereas odontogenic cysts and tumors, which frequently affect young adults, had the lowest mean age. Our results also indicate a significant association between gender and the occurrence of odontogenic cysts and tumors. Nevertheless, no significant correlation was observed between gender, age group, and the concordance between clinical and histopathological diagnoses, aligning with the findings of Farzinnia *et al*. (5) and Shamloo *et al*. (9) In contrast, studies by Forman *et al*. (6) and Soyele *et al*. (3) reported a higher concordance rate in women, while Tatli *et al*. (10) found a slightly higher discordance rate for lesions in female patients.

Reactive and inflammatory lesions were the most encourter lesions (35.1%), followed by epithelial pathology (10.02%), and immune-mediated lesions (7.58%), respectively. It is noteworthy that the prevalence of oral lesions in this study is based solely on biopsied cases and may not fully reflect the overall occurrence of oral lesions. A statistically significant correlation was identified between specific lesion types and clinicopathological concordance. Immune-mediated lesions demonstrated the highest concordance rate of 93.1%, likely due to their distinct clinical features and characteristic presentations. For instance, OLP often presents as a white, reticulated pattern known as Wickham’s striae (16,18). These findings align with previous studies, which reported high concordance rates (88.6%–100%) between clinical and histopathological diagnoses of OLP (5,7,19). In contrast, non-odontogenic cysts and tumors had the lowest concordance rate at 50%, presumably because they are relatively rare and their clinical features may resemble those of reactive and inflammatory lesions. A previous study by Mendez *et al*. (11) also reported a low rate of agreement for benign mesenchymal tumors, which were often misdiagnosed as reactive lesions.

The mandible was the most common biopsy site for oral and maxillofacial lesions in this study. This region is frequently associated with various odontogenic cysts and tumors, including dentigerous cysts, ameloblastomas, and odontogenic keratocysts. Among these, dentigerous cysts were the second most prevalent lesion observed. Consistent with the findings of Farzinnia *et al*. (5) and Shamloo *et al*. (9), our study identified a significant correlation between the anatomical location of lesions and the concordance between clinical and histopathological diagnoses. However, while both previous studies reported the highest concordance rates (5,9), the buccal mucosa exhibited the highest concordance in the present study. These discrepancies may be due to differences in sample sizes, lesion prevalence, and the level of clinical expertise across studies.

Importantly, when categorizing lesions as benign or malignant tumors, the concordance between clinical and histopathological diagnoses for malignant tumors dropped to 55.2%. This discrepancy likely occurs because the clinical presentation of malignant lesions can closely mimic various benign conditions, such as infection-induced osteomyelitis, periapical cysts, and other reactive or inflammatory lesions. Misdiagnosis can significantly impact both prognosis and the quality of life of patients. Early diagnosis improves the chances of complete recovery, reduces treatment-related side effects, and allows patients to resume their normal lives more quickly. Conversely, delayed diagnosis can result in more complex treatments and expose patients to both physical and psychological burdens. Notably, the type of biopsy was found to be associated with diagnostic concordance of malignant lesions, likely due to its selection based on lesion characteristics. Incisional biopsies are typically performed for large lesions or when malignancy is suspected, whereas excisional biopsies are more commonly used for smaller lesions or those initially diagnosed as reactive or inflammatory (20). Therefore, careful selection of the biopsy type is essential, as it can directly influence diagnostic accuracy and treatment planning.

## Conclusions

This study shows that approximately 70% of oral and maxillofacial lesions exhibit concordance between clinical and histopathological diagnoses. However, notable discrepancies remain in certain cases that need to be addressed. The degree of concordance can vary, as some lesions may present similar clinical features while having distinct histopathological characteristics. Since accurate diagnosis relies heavily on the clinician’s expertise, these findings underscore the importance of enhancing education, increasing awareness, and providing further training for dental professionals.

## Figures and Tables

**Table 1:** Age, gender, and concordance between clinical and histopathological diagnoses across lesion categories.

Lesion category	n (%)	Age (years)		p-value	Gender		p-value	Concordance (%)	p-value
		Mean	SD		Male	Female			
Reactive and inflammatory lesions	403 (35.10%)	41.54	20.39	<0.001*	181	222	0.210	233/403 (57.82%)	<0.001*
Epithelial pathology	115 (10.02%)	56.02	20.04	0.004*	37	78	0.219	77/115 (66.96%)	0.067
Bone pathology	69 (6.01%)	45.35	24.6	<0.001*	25	44	0.602	54/69 (78.26%)	0.157
Immune-mediated lesions	87 (7.58%)	53.48	12.49	0.867	26	61	0.333	81/87 (93.10%)	0.004*
Odontogenic cysts and tumors	378 (32.93%)	32.54	17.17	<0.001*	199	179	0.001*	301/378 (79.63%)	<0.001*
Non-odontogenic cysts and tumors	42 (3.66%)	39.34	19.91	0.148	22	20	0.351	21/42 (50.00%)	0.013*
Miscellaneous	54 (4.70%)	39.17	23.18	0.002*	23	31	0.494	29/54 (53.70%)	0.018*
Total	1,148 (100%)	40.97	20.70		513	635		796/1,148 (69.34%)	

* denotes statistic significant (*p* < 0.05), according to the Chi-square test.

**Table 2:** Concordance rate of clinical and histopathologic diagnoses based on age ranges.

Age range	n (%)	Concordance (%)	p-value
0-20	247 (21.50%)	175/247 (70.85%)	0.841
21-40	329 (28.70%)	223/329 (67.78%)	
41-60	331 (28.80%)	228/331 (68.88%)	
>60	241 (21.00%)	170/241 (70.54%)	

**Table 3:** Concordance rate of clinical and histopathologic diagnoses based on gender.

Gender	n (%)	Concordance (%)	p-value
Male	513 (44.69%)	348/513 (67.70%)	0.321
Female	635 (55.31%)	448/635 (70.66%)	

**Table 4:** Concordance rate of histopathologic and clinical diagnosis based on anatomical location.

Location	n (%)	Concordance (%)	p-value
Maxilla	290 (25.26%)	182/290 (62.76%)	0.001*
Mandible	320 (27.87%)	237/320 (74.06%)	
Buccal mucosa	150 (13.07%)	119/150 (79.33%)	
Gingiva and alveolar gingiva	191 (16.64%)	122/191 (63.87%)	
Tongue	54 (4.70%)	31/54 (57.41%)	
Palate	43 (3.75%)	32/43 (74.42%)	
Upper lip	15 (1.31%)	10/15 (66.67%)	
Lower lip	71 (6.18%)	55/71 (77.46%)	
Others	14 (1.22%)	8/14 (57.14%)	

* denotes statistic significant (*p* < 0.05), according to the Chi-square test.

**Table 5:** Age, gender, and concordance between clinical and histopathological diagnoses in benign and malignant lesions.

Lesions	n (%)	Age range				p-value	Gender		p-value	Concordance (%)	p-value
		0-20	21-40	41-60	>60		Male	Female			
Benign	1,119 (97.47%)	245	326	327	221	<0.001*	506	613	.024*	780/1,119 (69.71%)	0.094
Malignant	29 (2.53%)	2	3	4	20		7	22		16/29 (55.17%)	

* denotes statistic significant (*p* < 0.05), according to the Chi-square test.

## Data Availability

The datasets used and/or analyzed during the current study are available from the corresponding author.
